# Response to: RANZCP Position Statement 46: A missed opportunity to provide sophisticated guidance on asylum seeker and refugee policy

**DOI:** 10.1177/10398562251351439

**Published:** 2025-06-11

**Authors:** Suresh Sundram, Kym Jenkins, Tram Nguyen

**Affiliations:** Clayton, VIC; Malvern, VIC; Northcote, VIC

Dear Editor,

The article by Dr Spencer in December’s Australasian Psychiatry^1^ argues several issues are insufficiently considered in RANZCP’s Position Statement 46^2^ and if they had been, this would enhance the RANZCP’s reputation. According to Dr Spencer, ‘more extensive explication of reasoning and rebuttal of counter arguments’ was required. However, this is a misunderstanding of the function and intent of position statements. They are to state the RANZCP position and occasionally make recommendations. Position statements do not provide detailed, comprehensive expositions on complex matters.

Setting aside this misunderstanding, there are multiple major concerns; however, due to space constraints, two of which undermine the premise of the article are noted below.

The first is the questioning of evidence in the position statement about adverse health impacts of immigration detention. The Position Statement^2^ cites reports that describe directly the impact of detention on mental health in Australian controlled centres. External researchers are not permitted into centres; thus, these independent agency reports remain the best source of data. The one paper Dr Spencer quotes she does so erroneously citing the updated 2024 paper^3^ but quoting the earlier 2016 paper.^4^ If she had read the 2024 paper, she would have seen their conclusions are opposite to hers: ‘…all studies reported adverse effects on the mental health of detained asylum seekers’. It continues unequivocally: ‘…the use of detention should be discontinued altogether or reserved strictly as a last resort, justified by purposes beyond the mere status of being an asylum seeker’.^3^ This is consistent with the Position Statement.

Dr Spencer contends without any evidence that the entirety of the data across multiple countries, contexts, research groups and settings is potentially invalid because there may be secondary gain considerations. No evidence is presented to support her speculation.

Even more troubling is Dr Spencer’s selective presentation of data. Her succinct precis of immigration detention for unauthorised maritime arrivals details numbers since 1989 totalling 68,770 people. The Position Statement’s call for the release of these people into the community and an end to detention is the target of Dr Spencer’s argument. She contends that community concern and cohesion is vulnerable and should be incorporated into the Position Statement. The implication is that, *inter alia*, community cohesion, housing, jobs, infrastructure and health systems are threatened and crime risks are plausible outcomes from releasing people from detention. What Dr Spencer fails to mention is that during the briefer period from 2001 to 2023, some 2.7 times as many, that is 188,775 other people, sought asylum in Australia.^5,6^ These people arrived by plane and almost all lived in the community whilst their claims were being processed. These claims were processed and these people did not in any evidenced way uphold Dr Spencer’s concerns. Critically this argues that detention is unnecessary for the routine processing of asylum claims in Australia and that the Australian community is able to accommodate people at far higher numbers than those that concern Dr Spencer.
